# Co‐Production Experiences in Practice From Funding to Implementation: Co‐Production Experiences of Academics and Consumers in Real‐World Practice

**DOI:** 10.1111/hex.70588

**Published:** 2026-03-26

**Authors:** Samantha Kozica Olenski, Anusha Ramani Chander, Georgina Chambers, Leslie Arnott, Deborah Loxton, Michelle Peate, Helena Teede

**Affiliations:** ^1^ Monash Centre for Health Research and Implementation Monash University and Monash Health Clayton VIC Australia; ^2^ Centre for Big Data Research in Health and School of Clinical Medicine University of New South Wales (UNSW Sydney) Sydney NSW Australia; ^3^ Centre for Women's Health Research, College of Health, Medicine and Wellbeing The University of Newcastle Newcastle NSW Australia; ^4^ Hunter Medical Research Institute Newcastle NSW Australia; ^5^ Department of Obstetrics, Gynaecology and Newborn Health Royal Women's Hospital, University of Melbourne Parkville VIC Australia

**Keywords:** consumers, co‐production, partnerships, women's health

## Abstract

**Background:**

Consumer and community involvement in health research is increasingly recognised as essential for ensuring relevance and responsiveness to diverse population needs. The Australian Women's Health Research, Translation and Impact Network (WHRTN) aims to improve women's health and build capability in women academics. WHRTN awarded targeted co‐production grants to support collaborations between academics and consumer teams across women‐centred research priority areas. This study explores the application of co‐production processes, including the experiences of academic and consumer leads in planning and implementing projects in real‐world research.

**Methods:**

Qualitative semi‐structured interviews were conducted with academic and consumer leads. Template analysis was used to thematically and deductively code de‐identified transcripts, guided by the Consolidated Framework for Implementation Research and the National Institute for Health and Care Research co‐production principles.

**Results:**

Twenty interviews revealed that co‐production was operationalised in diverse ways to meet contextual needs and deliver meaningful outcomes. Consumer and community involvement were consistently prioritised, with principles such as power sharing, equality, reciprocity, and relationship‐building evident. Project progress and outcomes were influenced by the developmental stage of the research team, project maturity, and strength of existing partnerships. Key enablers included strong leadership, prior co‐production experience, mutual respect, open communication, established networks, and clear project goals. System and organisational challenges included the slow pace of co‐production, resource demands, and working in sensitive areas such as Indigenous health and sexual violence.

**Conclusion:**

The value of involving consumers in project planning and implementation to optimise impacts is clear and is supported by strong leadership, a culture of mutual respect and a clearly defined project scope. However, system and organisational barriers to meaningful co‐production persist. Recommendations to enhance co‐production include multi‐stage funding processes, policy and organisational changes, expanded opportunities for co‐production training, and allocation of additional project funded time. Transparent sharing of co‐production processes and learnings is needed to embed co‐production into routine research practice.

**Patient or Public Contribution:**

Each funded project was jointly led by academics and consumers. Consumers contributed to all stages of project design and implementation, ensuring their perspectives informed decision‐making. An experienced consumer partnership lead contributed to data interpretation and preparation of this manuscript and is a listed author of this work.

**Abbreviations**
AHRA: Australian Health Research Alliance
CFIR: Consolidation Framework for Implementation Research
NIHR: National Institute for Health and Care Research
WHRTN: Women's health research translation and impact network

## 1 Background

Increasingly the involvement of consumers and community consultations is being recognised internationally as a cornerstone in the planning and delivery of health research and practice. The Australian National Health and Medical Research Council (NHMRC) defines consumers as ‘patients, potential patients, carers, and current or potential users of health services, including individuals or organisations representing their interests’ [1]. Involving consumers’ expertise is crucial to ensure that research is relevant, responsive, and effectively addresses the needs and preferences of diverse community populations [2, 3]. Co‐production is a consumer and community consultation approach where researchers, practitioners, and consumers work together from the beginning of a project, to generate new knowledge of a health condition or issue together [4]. Co‐production is an inclusive, participatory approach grounded in principles of equity, trust‐building, partnership development, power‐sharing, rigorous evaluation, and transparent governance processes necessary to enhance the impact of research [2]. However, embedding co‐production in research remains challenging and there is limited practical knowledge of processes required to guide ‘what’ and ‘how’ to best implement co‐production [3, 4, 5]. Continuous evaluation and documentation of how co‐production is implemented in real world settings is important to help guide best practice and to forecast the direction of future research [3, 4, 6].

In Australia, efforts to integrate consumer and community involvement (CCI) into health and medical research has intensified and it is now a requirement within national grant funding policies. The NHMRC's 2016 ‘Statement on Consumer and Community Involvement in Health and Medical Research’ co‐authored by the Consumers Health Forum of Australia reflects commitment to strengthen researcher academics capacity to undertake meaningful community and community engagement [1]. Furthermore, Co‐production is also a priority area for the [7], who are a collective of 12 government funded and recognised accredited Research Translation Centres, focused on integrating research into healthcare [7]. (Consumer and Community Involvement).

The Women's Health Research, Translation and Impact Network (WHRTN) is a national network established under the auspices of AHRA in 2018 and funded by Medical Research Future Fund in 2020 [8]. The WHRTN network aims to improve the health of women by integrating prevention, healthcare, research, and translation activities and to build capability in women researchers across the AHRA networks. There are nine key areas of women's health prioritised by WHRTN, aligned to the National Women's Health Strategy 2020‐2030 and developed in consultation with community members. These include preconception, pregnancy, postpartum and intrapartum health of women and babies; mental health; reproductive health; chronic disease and preventative health including cancer and heart disease; healthy lifestyle, nutrition, physical activity and the prevention of obesity; violence and abuse; Indigenous health; healthy ageing; and sexual health [8].

In 2022, WHRTN and AHRA awarded nine co‐production project grants through a two‐step process, aligned with WHRTN's priority research areas. These grants aimed to strengthen collaboration between academic and consumer teams across AHRA Centres, reinforcing CCI as a system‐level priority.

The objectives of this study were as follows:

1. To examine how co‐production methods and principles were applied across the diverse WHRTN‐funded projects and priority areas.
2. To explore academic and consumer grant recipients’ experiences and insights in applying co‐production methods during the WHRTN‐funded projects.

## 2 Methods

### 2.1 WHRTN Co‐Production Grant Scheme Design

The WHRTN co‐production grant scheme embedded requirements for consumer involvement from initial development through to implementation and evaluation. Consumers contributed to designing the grant scheme, including determining the application guidelines and assessment criteria, and participation in the peer review process of submitted applications. Grant applications were assessed against pre‐specified community priorities in women's health research, with a review panel consisting of WHRTN Steering Committee members and external reviewers, including at least one consumer representative per application. The assessment criteria for grants included: alignment with WHRTN's priorities for women's health research determined via a large consumer survey (equity, innovation, and innovation), transparency, co‐production methodology and budget, and the overall capacity of the team. The co‐production project proposal needed to reflect the National Institute for Health and Care Research (NIHR) Guidance on Co‐producing principles for research projects [2]. This guide is part of the NIHR first strategic commitment for public partnerships to improve how they work with patients, service‐users and carers. The key principles highlight; (1) the importance of the sharing of power, (2) including all perspectives and skills, (3) respecting and valuing the knowledge of all those working together, (4) reciprocity, and (5) building and maintaining relationships [2].

**Figure 1:**
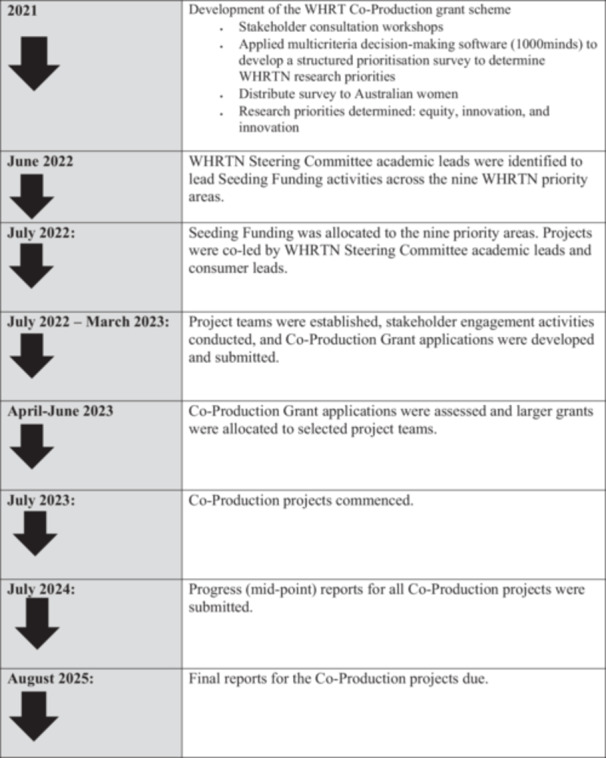
Seed funding and co‐production grants scheme and distribution process and timing. Illustrates the sequential stages of the seed funding and co‐production grants, highlighting key phases and their timing across projects timelines.

The co‐production project funding was allocated in a two‐step process. Initial ‘seed’ funding was allocated in 2022 to WHRTN steering committee member/s who partnered with a consumer lead to form project teams. This team collaborated during the 6‐month seed grant period to develop a project grant application for the full co‐production project. Each co‐production project grant application required a single academic lead investigator responsible for delivering the research programme for a maximum duration of 2 years. Applications could include up to ten co‐investigators: five academics, including at least one early‐ or mid‐career researcher, and five consumer investigators. For a detailed overview of the grant scheme and distribution process see figure 1.

To support the implementation of NIHR Co‐production principles, successful grant applicants received online co‐production training delivered by WHRTN's experienced Consumer Partnership Lead. In addition, a detailed resource package was provided, which outlined co‐production principles and included further training materials for both consumer and academics investigators. Ongoing project monitoring was undertaken to ensure equitable consumer partnerships across the duration of the projects.

### 2.2 Sampling and Recruitment for Interviews

We aimed to separately interview all co‐production ‘seed’ and/or fully funded research grant leads and consumer leads during 2023‐2024 when the funded projects were being undertaken. All co‐production grant recipients (academic and consumer leads) were invited to participate, via email from the WHRTN Chief Investigator. All participants received information regarding their involvement and consumers leads were remunerated with gift cards.

### 2.3 Qualitative Data Collection, Theoretical Framework and Analysis

The semi‐structured interview guide was informed by the Consolidation Framework for Implementation Research (CFIR) (Appendix 1). The CFIR was developed to guide systematic assessment of multilevel implementation contexts to identify factors that influence implementation. It comprises of 5 key domains; Innovation (focused on understanding the nature of the intervention), Outer setting (focused on understanding the external influences), Inner Setting (focused on understanding the project team), Individuals (focused on understanding the characteristics of leaders and team members), and Implementation Processes [9, 10]. The categorisations within CFIR offer a robust structure for examining the challenges and facilitators for implementing co‐production principles and practices. Additionally, the NIHR principles were applied to explore co‐production methodologies, particularly the roles and experiences of consumers [2].

Two experienced female qualitative researchers completed the research and had no prior relationship with interviewees. The research was supervised by the investigator team. Semi‐structured interviews were conducted after obtaining informed consent. Interviews were audio recorded, de‐identified and transcribed, and field notes maintained. Template analysis was undertaken to thematically and deductively code transcripts according to the CFIR key domains [10], enabling the identification and categorisation of primary patterns within the data. Template analysis is a flexible process in which new information can be easily incorporated or adjustments made [11, 12]. Development of the final template is an iterative process in which modifications are possible and expected [11]. As part of this process, an initial coding template based on preliminary analysis of all interviews was developed. In‐depth discussion of emerging themes was conducted amongst study investigators before a final iteration of the results. This was finetuned with guidance from an experienced investigator group. Analysis and development of models was assisted by the NVivo Software programme (QSR International Pty Ltd. Version 14, 2024, Victoria, Melbourne). Verbatim quotes from interviews that best represented the key findings for each theme were highlighted for subsequent reporting purposes.

This study was reported according to the recommendations of the Consolidated criteria for Reporting Qualitative studies (COREQ) [13].

## 3 Results

Following the development and approvals of the nine co‐production grant applications using ‘seed’ funding, the full Co‐production Grants were allocated in mid‐2023 with projects to be completed by July 2025. Grant recipients lived and worked in the states of Victoria, South Australia, New South Wales, Queensland and Western Australia and projects represented a broad spectrum of health areas and disciplines (Table 1).

**Table 1:** Overview of co‐production projects across the nine identified research priority areas.

Priority area	Topic area	Aims	Methods	Co‐production application and the role of consumers	Anticipated outcomes
Mental health	Perinatal mental well being for refugee and migrant women	Improving access to culturally responsive information on perinatal mental wellbeing for women from refugee and migrant backgrounds	Scoping review and mapping of digital resources Interviews with women and resource developers/managers Workshops	Consumers involved in topic selection and all phases of research Co‐lead meetings Used personal networks to recruit women for interviews Translation of flyers and other recruitment materials	Knowledge generation Framework to support co‐design of digital resources for vulnerable women
Preconception, pregnancy, postpartum	Weight‐inclusive maternity care	To describe larger‐bodied women's experiences with weight‐inclusive care during preconception, pregnancy, birth, and postnatal periods. Produce best practice principles for weight‐inclusive care.	Interviews Discussion forums and workshops Surveys Photovoice study	Topic selection Facilitated focus group Chaired consumer meetings Key contact person for the consumer group	Knowledge generation Awareness raising Consensus on best practices
Reproductive health	Elective egg freezing (EEF) information needs	Identify information needs of women interested in EEF. Develop a consumer web‐based resource.	Literature review Interviews Quantitative modelling Google analytics Surveys	Involved in project development and topic selection Survey design and questions	Knowledge generation Web‐based resource development
Chronic disease and prevention	Chronic disease prevention in women	Promote the use of chronic disease screening. Assess acceptability of web‐based resource for screening female cancers, bowel cancer and chronic diseases	Partnership building Online forum discussions Focus groups.	Topic selection Co‐chaired meetings Online forum	Knowledge generation Awareness raising Web‐based resource development
Healthy ageing	Social connections for midlife women	Raise awareness of the health benefits of social connectedness. Develop resources for community centres and train volunteers to deliver support.	Knowledge review Roundtable discussions and priority setting Podcast development.	Topic selection Lead workshops to define topic area Received formal co‐production training Initiate design and development	Knowledge generation Community support resources To train volunteers to support program delivery
Healthy lifestyle, nutrition, physical activity and the prevention of obesity	Heart health for midlife women	Identify factors affecting heart health in midlife women. Evaluate evidence on CVD knowledge and prevention behaviours. Explore needs and tools for lifestyle change. Co‐design and evaluate a heart health literacy initiative.	Systematic review Needs analysis survey Interviews Usability testing	Topic selection Key role in exploring existing available resources Involvement in resource development	Knowledge generation Co‐designed initiative for CVD prevention
Indigenous health	Disordered eating in indigenous communities	To co‐design and evaluate a culturally appropriate eating disorder model of care and health professional training module.	Partnership building, surveys, interviews.	Topic selection Contributed to interview and survey questions	Knowledge generation Improved access to culturally competent care Eating disorder training model development
Sexual health	Sexual health for LGBTQ women with disabilities	Examine the intersection of LGBTQ identities and disability in sexual health. Identify facilitators and barriers to culturally safe sexual healthcare.	Surveys Interviews Arts‐based methods (body‐mapping and photovoice)	Community engagement Development terms of reference Drawing on existing networks	Knowledge generation Healthcare policy support
Violence and abuse	Trauma‐informed healthcare	Provide insight into women's experiences with health services to identify gaps in trauma‐informed care. Understand health providers’ knowledge, experiences and develop recommendations to improve care	Focus groups Surveys	Contributed to topic selection and refinement Survey and focus group questions Participant recruitment Project reporting	Knowledge generation Consensus recommendations for best practice trauma‐informed care

### 3.1 Semi‐Structured Interviews

Twenty interviews were conducted between May to October 2024 with co‐production grant recipients, both academic and consumer leads.

#### 3.1.1 Academic Leads

12 personalised email invitations were sent out and 11 interviews were completed; Of note, 12 recruitment emails were sent out for the nine projects, as some priority projects had different seed and fully funded grant leads. All academic leads held university positions of A/Professor or Professor or Research Fellow, and reported significant experience in co‐design and working with consumers. On average academic leads had been working with consumers for 18 years (range of 6–30 years). Interviews ranged from 30 to 57 min with the average length being 40 min.

#### 3.1.2 Consumer Leads

9 personalised email invitations were sent to consumer leads and 8 interviews were completed. One consumer lead was not available. Consumer leads had diverse professional roles in their community including health, public sector and not‐for‐profit employees, as well as privately owned business owners. All consumer leads reported that they had worked in consumer advocacy and/or co‐design activities, with an average time of 11 years participating in consumer roles (range 4‐24 years). The interviews ranged from 20 to 48 min, with the average length 34 min.

**Figure 2:**
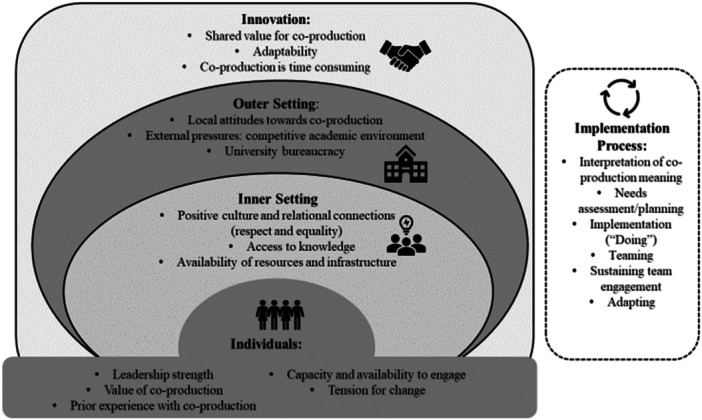
Summary of constructs and themes impacting co‐production project. outlines the summary of constructs and themes impacting co‐production projects, with themes mapped to CFIR domains (in, outer setting, inner setting, characteristics of individuals, and process).

Results are presented by CFIR domain, with identified themes mapped to the relevant CFIR constructs (Figure 2). Overall, academic and consumer reflections and experiences were aligned across the CFIR domains with minimal differences of note.

Table 1 summarises the nine WHRTN projects. Exemplar quotations pertaining to themes are included in Appendix 2. Please note assigned quote numbers do not reflect the order of grants listed in Table 1.

### 3.2 Innovation

#### 3.2.1 Relative Advantage and Adaptability

All academics and consumers strongly valued co‐production recognising its importance in health research. Academics highlighted their openness to sharing power with consumers, a sentiment agreed and echoed by consumer participants and consistent with the NIHR co‐production principles. One of the key advantages of the grant was that it was not prescriptive offering the study teams flexibility to adapt data methods to consumer needs and context. The initial co‐production ‘seed’ grants included a priority setting activity to determine the project aims, target audience and implementation plan for developing the full grant application. A diverse range of methods were utilised during the ‘seed’ funding period to inform the development of the fully funded grant application. For example, the weigh‐inclusive maternity care project (Priority Area, Preconception, pregnancy, postpartum) engaged larger bodied women and weight‐inclusive clinicians to find acceptable and feasible ways to capture their perspectives on living well during pregnancy, birth and postnatal care. Here, a photovoice study was undertaken whereby women utilised a closed website to share photos and images symbolising their experiences and engage in asynchronous online discussions. While the Indigenous and Sexual Health teams, utilised flexible data collection methods such as community interviews, surveys and visualisation boards to enable vulnerable community members to be heard during topic selection. A visualisation board is a collaborative and culturally grounded tool to support discussion, reflection, and shared understanding between researchers and community members. This method allows participants to place images, drawings, words, symbols, or artefacts that represent their experiences and health priorities. In contrast, the Reproductive Health team utilised Google Analytics to ascertain what information consumers were most searching for in reproduction health (Table 1). These participatory and data‐driven approaches were designed to foster meaningful engagement and to capture a breadth of consumer perspectives, to ensure consumer priorities for research were captured.

### 3.3 Design and Cost

Consumers were actively engaged as partners across all stages of the research, contributing to topic selection, study design, recruitment, and implementation (Table 1). Their numerous roles included co‐leading meetings and workshops, supporting community engagement, drawing on existing personal networks for community engagement, conducting focus groups, designing and reviewing resource, and translating recruitment materials.

While the value of co‐production was universally recognised, academics frequently reported substantial logistical and administrative complexities. Co‐production was described as resource intensive with significant coordination, time, and financial investments required.

Co‐production definitely takes time, is not a quick process… but what we do have is a really strong consumer‐led or consumer‐engaged approach, and we will have really rich data.

(Academic lead #3)

Consequently, co‐production was perceived by many as a slower and more expensive approach compared to conventional research methods. Several academic leads reported that the allocated grant funding and time (2.5‐years) was insufficient to support the level of engagement and involvement required to genuinely coproduce with consumers. The broad scope of WHRTN's priority areas made consensus on topic selection particularly challenging, contributing to frustration and delays in project initiation for some teams.

The timeline to get it all done was a lot to get to. And like it would be okay that timeline if you had preconceived ideas about what your grant was going to be and you followed those. If you were going from a true co‐production standpoint and starting from square one, it was a lot.

(Academic lead # 3)

Importantly, the grant allowed consumers to be reimbursed for their time and contribution included travel expenses. This was immensely valued by consumers and academics.

The privileging of the consumer voice—making sure that we remunerate consumers appropriately for their time, that the engagement is authentic and robust, and that their contribution is acknowledged at every stage of the pipeline.

(Academic lead #4)

### 3.4 Outer Setting

#### 3.4.1 Financing, Partnerships, External Pressures and Local Attitudes

Although coproduction has been identified as a priority by Australian grant funders, WHRTN funding initiative was widely regarded as novel, given the limited availability of dedicated co‐production funding opportunities in Australia. The relatively less competitive nature of the WHRTN initiative enabled researchers to focus on co‐production without the typical pressures and constraints associated with traditional highly competitive research funding models. Additionally, many teams were able to leverage strong pre‐existing partnerships with community organisations, local councils, and healthcare services, which facilitated stakeholder engagement for seamless project initiation and implementation.

Conversely, several external pressures impeded the implementation of the co‐production projects. Within the highly competitive academic landscape in Australia, researchers frequently reported significant challenges related to time constraints and competing professional obligations. Demands such as grant writing, manuscript preparation, team and student supervision, and broader institutional responsibilities limited, in some cases, their capacity to fully engage in the time‐intensive processes of co‐production. The environment makes it ‘difficult to pause, reflect, and meaningfully incorporate diverse perspectives’ (Academic lead #10), thereby impeding the collaborative nature that underpins effective co‐production.

If you were focused on the papers…how are we going to get the next grant, we are thinking about that obviously. You compromise your ability to do the engagement well.

(Academic lead #1)

Some interviewees described that CCI was not always rewarded within traditional academic structures, often perceived as *‘*soft research’ (Academic lead #1) which impacted academic institutional support and prioritisation. Bureaucratic barriers such as delays in ethical approvals and administrative processes for funds distribution were also reported to slow progress particularly for set up of projects addressing sensitive research priorities, including Indigenous health, sexual health, and violence and abuse. These topics brought inherent ethical and cultural complexities, adding further layers of challenge to project initiation and implementation.

### 3.5 Implementation Process

#### 3.5.1 Assessing Needs and Context

The distinction between co‐design and co‐production was discussed. Co‐design was viewed as a process that primarily engages consumers during the early stages of a project, with consumer input tapering thereafter and academic teams maintaining dominant control over decisions and implementation processes. and. In contrast, co‐production was perceived as a process that ensures continuous collaboration throughout all project phases, with consumers playing an equal role to academic in decision‐making and implementation. Key differences described include the nature of collaboration, timing of involvement, frequency of engagement, and the shift of power toward more equal partnerships in co‐production, with shared decision‐making a defining feature.

Co‐design is involving consumers in the work but co‐production really is that equal lead or sharing the lead throughout the project, which is different.

(Academic lead #6)

Anticipated project outcomes of the full project grants included the development of podcasts, training modules, and digital tools; the co‐creation of consumer resources and evidence‐based practice guidelines; the establishment of expert and consumer reference panels; and dissemination through publications and conference presentations (Table 1).

### 3.6 Planning

Both implementation strengths and challenges were reported. Narrowing down the project scope and clarifying roles emerged as key themes for effective implementation and meaningful consumer involvement within the constraints of the allocated project funding timelines. Early clarification of these two factors, fostered collaboration, established shared expectations, and improved team functioning. In a small number of projects, poorly defined roles and unclear grant expectations, resulted in delays for teams when selecting a topic, delaying project progress.

### 3.7 Teaming

Teams with prior partnership history were able to quickly establish project topics and were better positioned to efficiently transition to the 2‐year full project grant period. Academics and consumers alike highlighted that identifying appropriate partners, securing commitment from various collaborators is ‘difficult’, noting that it takes considerable time to bring people together, build effective partnerships and form consensus for research priorities.

The challenge is getting very busy people, very senior people, and people who are wearing a lot of hats into a room.

(Academic lead #5)

Without sufficient early investment in team formation and cohesion, projects were more likely to experience persistent delays, and miscommunication, likely to affect project progress. A third of teams reported challenges in identifying and engaging appropriate consumer representatives. Academic leads noted that, given consumers often have multiple competing personal and professional demands, maintaining their involvement over a 2‐year co‐production project was difficult, leading to several discontinuing their involvement.

We have had some of our consumer team step back just because everyone is a parent and have lives and jobs. (Consumer lead, #5)

### 3.8 Inner Setting

#### 3.8.1 Culture and Relational Connections

Overall, all consumers agreed that the principles outlined in the NIHR Guidance in Co‐Producing a Research Projects [2] were successfully applied. Interviewees consistently described the importance of a strong culture of respect, reciprocity, trust, empowerment, and open communication to support coproduction processes.

We (feel) validate respected because our perspectives are always taken on board. Like it was not like only, okay, you are talking so we'll listen to you and it was equal, equitable… we felt safe and respected.

(Consumer Lead #4)

Consumers reported feeling genuinely valued and listened to, reflecting experiences of authentic collaboration rather than tokenistic involvement, something many had encountered in prior research involvement. All academic leads described positive relationships with their consumer leads and strongly valuing their relationship. This aligned with most consumers’ views that the development of strong, open relationships was foundational to equal partnership and to ensuring that their voices contributed to the research. However, a smaller number of consumers acknowledged that, despite best efforts, an underlying power imbalance between academics and consumers is inevitable due to academic's expertise being more experienced in undertaking academic research.

Importantly, all emphasised the value of grants that centre the consumer experience. Many consumers described personal and professional growth resulting from their involvement, particularly among those who had received training in co‐production methods, this is in keeping with NIHR principle of reciprocity [2].

The benefits are probably intangible but can contribute to professional knowledge and also personal knowledge.

(Consumer Lead, #2)

### 3.9 Access to Knowledge

A consistently reported facilitator of successful implementation was the presence of knowledgeable, high‐quality project team members. Many academic leads described teams composed of individuals with diverse skill sets and professional experiences, which enriched the co‐production process. In particular, experienced members played a vital role in guiding project development and providing opportunities for consumer training and support. As one academic lead reflected,

So I think part of the success is that it is a team of women who go above and beyond and make the budget really stretch.

(Academic lead #1)

Consumers, highlighted the importance of co‐production training for both academics and consumers to strengthen involvement and support meaningful, active participation and, noted that additional provision of training would have been useful.

In hindsight, I think I would like to have had a better understanding of what co‐production actually meant (at the outset).

(Consumer Lead #6)

### 3.10 Infrastructure and Resources

Project leads who reported substantial progress commonly attributed their success to access to sufficient resources and availability of infrastructure, including dedicated project teams, funding, and in‐kind institutional support. Many described employing creative strategies to extend their resources, such as leveraging research centre funds, professional fellowships, or securing additional small grants. In contrast, those with limited resources and staff commonly reported delays in project commencement and progress.

### 3.11 Individual Setting

#### 3.11.1 Leadership

Experienced leadership was identified as a key facilitator of successful co‐production. Academic leads with extensive research and co‐production experience were often able to refine the project focus, scope and navigate complex decision‐making. Notably, projects required motivated leaders to ‘do the heavy lifting’ and see through the project implementation. In projects where leadership and co‐production experience were less established, progress was slower and broader team engagement more variable

Similarly, strong consumer leadership was also reported as integral to fostering a culture of trust, reciprocity, and collaboration. Consumer leads with prior research experience, deep community insight, strong personal networks, and high levels of motivation were particularly effective in supporting recruitment and driving implementation.

### 3.12 Tension for Change

While all academics strongly recognised the value and input of consumers, many acknowledged that the shift in power dynamics and changes to traditional research methods could be challenging. Some challenges described related to a *‘tricky balance*’ of hearing suggestions that were valued but often would likely lead to project delays, additional resources and extending timelines.

It's been hard in the sense of I've really had to change the way I do things. It's a psychological shift.

(Academic lead #11)

Despite some tensions related to change, both academics and consumers reported high levels of satisfaction with their participation in the WHRTN co‐production grants. All, especially mid‐career researchers highlighted the grant as an important opportunity to build their co‐production knowledge, develop networks with researchers outside their institutions, and gain access to mentoring and leadership experience.

## 4 Discussion

This study demonstrates that co‐production principles and methodologies were applied in diverse ways across nine WHRTN‐funded projects, reflecting flexibility in implementation tailored to specific research contexts. Across all settings, consumer and community involvement was consistently prioritised, with core principles such as power sharing, equality, reciprocity, and relationship‐building clearly evident in line with NIHR principles to guide co‐production. Application of the CFIR framework helped identify that individual academic and consumers, operate within a project team (inner setting) and are also influenced by the broader outer environment (including universities and funding bodies). Therefore, factors that supported successful co‐production in addition to individual skills and motivation, included strong leadership, prior experience with co‐production approaches, a culture of mutual respect, open communication, existing community or professional networks, role clarity, and clearly defined project scope and goals.

Project progress and anticipated outcomes were influenced by the developmental stage of the research team, the maturity of the project concept, and the strength of pre‐existing partnerships. Attempts to determine the project topic through consensus methods during the initial ‘seed’ phase led to some frustration and delays, given the significantly broad nature of the WHRTN priority areas. Without sufficient time and early investment in team formation, cohesion and topic selection, projects were more likely to experience persistent delays, miscommunication, and reduced overall project progress. Improvements are needed to better support this early‐stage process in future rounds and could be achieve by ensuring, multi‐stage planning process, beginning with consumers involvement in selecting broad principals and priority areas, and aligning with national health strategies. Additional challenges included the significant time and resources required to establish and maintain teams, sustaining consumer involvement over extended periods, and working in sensitive areas such as Indigenous and Sexual Health. Despite these challenges, academics and consumers reported high satisfaction with the co‐production experience. Improvements focused on greater co‐production training for both consumers and academics.

To the best of our knowledge, this is one of the few grant initiatives focused primarily on co‐production that reflectively explores the experiences and learnings of both academics and consumers. These findings offer practical insights into the operationalisation of co‐production in real‐world research settings, a gap identified by two recent rapid reviews. These reviews emphasise that the lack of comprehensive reporting limits opportunities for knowledge exchange and hinders the ability of others to effectively integrate and operationalise co‐production principles in their own practice [4, 14].

A key theme emerging from our analysis, was the distinction between co‐design and co‐production. The use of inconsistent terminology to describe the development of initiatives involving multiple stakeholder including consumers has been established as a critical barrier to strengthening co‐production efforts [3, 15, 16]. This highlights the need to streamline definitions in order to create transparency, build shared understanding, funding guidelines and reduce ambiguity in practice [17]. A key strength of the grant funding model was the flexibility, which enabled teams to allocate resources in accordance with their specific needs, priorities and contexts. The importance of adapting co‐production methods to suit diverse contexts is well supported in the literature [4, 18].

In line with previous studies, we also identified facilitators and skillsets to support co‐production that include: strong partnership building skills with diverse stakeholders, managing roles and expectations, open and flexible communication, and more time required than conventional research [4, 18]. These facilitators align with the principles outlined in the NIHR guidance for Co‐Producing a Research [2], and were evident across most participating projects. Consumers consistently emphasised the importance of feeling like equal partners, experiencing genuine shifts in power, and being meaningfully valued. This stands in contrast to much of the existing literature, which frequently describes delayed or symbolic consumer involvement as indicative of tokenism and a lack of authentic collaboration [19]. However, also consistent with the literature, academics acknowledged that the shift in power balance could be challenging [14].

Despite growing interest in co‐production nationally and internationally, critical challenges remain in its implementation at organisational and systems levels [3]. In our study, academics identified persistent ‘outer‐level’ barriers, including time constraints and competing professional demands within the competitive academic environment, which limits their capacity to engage meaningfully in co‐production. Academic leads reported that these pressures, coupled with a lack of institutional recognition of co‐production, reduced organisational support and prioritisation. These findings align with prior literature recognising that effective co‐production requires changes in academic institutional practices, to support more equitable power sharing in research processes [3, 20, 21]. However, achieving this remains challenging, as health and social care research is often conducted within entrenched hierarchies and structural inequalities embedded in universities, public service institutions, and research funding systems [5]. Both consumers and academics highlighted the need for greater opportunities for co‐production training, including access to structured supports, as well as availability of evidence based frameworks, tools and resources. Some of the authors in this initiative have led a national programme for promoting best practice and consumer and community involvement. This involves resources training and standard operating procedures for academics and consumers, and implementation guide for organisations and influencing national funding policy. This should help address some of these concerns [22].

Our findings highlighted that short timelines and rigid structure within traditional funding models constrain early‐stage collaboration, particularly by limiting time and resources needed to build partnerships across institutions, organisations, and consumers. Limited funding opportunities for co‐production remains undoubtably a key barrier and could be overcome by allocating funding and resources in the planning stage and having adequate resources to ensure inclusivity and reciprocity throughout the process [4, 23]. This would also mitigate the persistent challenge of sustaining consumer involvement beyond the project period, including contributions to post‐project outputs such as publications and presentations. Sustaining relationships between researchers and trained consumers outside active funding cycles are essential, yet challenging. Consistent with the literature, to promote optimum and impactful co‐production we highlight the need for changes to funding structures, opportunities for knowledge gains and capacity building, equitable engagement and transparent reporting [3]. Finally, extending funding timelines would provide the necessary space to build relationships, plan collaboratively, and ensure authentic co‐production processes.

## 5 Limitations

The study's findings should be interpreted with consideration of limitations. Some of the academic leads interviewed were both project leads and co‐authors of this manuscript, which may introduce potential bias. However, they were included due to their substantial involvement in WHRTN and considerable expertise in this area. Although interviewers were external to the project teams, consumers may have felt a perceived obligation to report positive co‐production experiences. Additionally, only the academic and consumer leads were interviewed, meaning perspectives from other team members were not captured. Interviews with WHRTN steering committee and project administrative staff will be reported in subsequent publications. Finally, data collection took place during the grant period to explore processes as they unfolded; therefore, complete project outcomes were not yet available. A follow‐up analysis focused on outcomes will be conducted upon project completion.

## 6 Conclusion

Academic and consumers operationalise co‐production in various ways to address contextual needs and to provide meaningful outcomes. Implementation of co‐production projects were optimised by a culture of mutual respect, open communication and narrowing down the project topic. This study highlights that, although there is growing recognition of the importance of applying co‐production principles throughout the research process, barriers to implementation extend beyond the capabilities and motivation of individual academics and/or consumers. Systemic and organisational barriers also play a significant role in influencing the feasibility and success of co‐production efforts. Co‐production undertaking requires a multi‐stage process characterised by consumer involvement in all phases. This begins with collaborative priority setting and progresses through the refinement of project scope to ensure alignment with team expertise, resource availability, and the constraints of funding timelines. Recommendations to enhance co‐production include multi‐stage funding processes, policy changes and organisational supports to foster a culture of respect and power sharing, greater opportunities for co‐production training, promotion of transparent reporting of learnings, and allocation of more funded project time.

## Author Contributions

All authors contributed to the conceptualisation of the manuscript and project activities. Samantha Kozica‐Olenski, Helena Teede and Anusha Ramani‐Chander led the drafting of the manuscript, with substantive input from all authors. Georgina Chambers and Helena Teede developed the co‐production grant scheme. Leslie Arnott provided a consumer advisory perspective for interpreting interview data and manuscript writing. Michelle Peate and Deborah Loxton contributed their grant experience and assisted in framing key learnings. All authors read and approved the final manuscript.

### Ethics Statement

This study was approved by the Monash Health Human Research Ethics Committee (HREC/73642/MonH‐2021‐274115). All participants gave consent to participate.

### Conflicts of Interest

The authors declare no conflicts of interest.

## Data Availability

The data that support the findings of this study are available on request from the corresponding author. The data are not publicly available due to privacy or ethical restrictions.

## Appendix 1 Interview schedule questions mapped to Consolidated Framework for Implementation Research (CFIR)

1. Innovation/Intervention Characteristics
  - Focus: Understanding perceptions, definitions, and design of co‐production interventions.
    ◦ Intervention Source/Evidence Strength & Quality
    ◦ How did you learn about co‐production?
    ◦ What key evidence or online resources did you use to support your co‐production practice?
    ◦ Adaptability/Complexity/Design Quality and Packaging
    ◦ How did you define co‐production in your project?
    ◦ What principles or key elements guided your co‐production approach?
    ◦ What methods did you use and why?
    ◦ How did the approach change or adapt over time?
2. Outer Setting
  - Focus: External context, stakeholder needs, and influence of broader systems.
    ◦ External Policy & Incentives
    ◦ How does your project align with WHRTN co‐production priorities: healthcare improvement, equity, innovation?
    ◦ What influence did policy, funding criteria, or system‐level expectations have?
3. Inner Setting
  - Focus: Organisational environment, CULTURE and support for implementation.
    ◦ Implementation Climate/Culture/Readiness for Implementation
    ◦ What resources (e.g., personnel, organisational support) were available for planning and conducting co‐production?
    ◦ What internal factors helped or hindered your co‐production efforts?
    ◦ Networks and Communications
    ◦ Who is part of your co‐production network, and how does it function?
    ◦ How do you communicate with your network (e.g., virtual/in‐person)?
4. Characteristics of Individuals
  - Focus: Experience, beliefs, knowledge, and relationships.
    ◦ Knowledge & Beliefs about the Intervention
    ◦ What prior experience did you have with co‐production?
    ◦ How has your understanding of co‐production evolved?
    ◦ Self‐efficacy/Individual Identification with Organisation
    ◦ What has been your experience working with consumers/academic lead?
    ◦ How do you perceive the consumer‐researcher relationship?
    ◦ What role does the consumer lead play, and how did this relationship develop?
5. Process
  - Focus: Planning, executing, evaluating, and sustaining implementation.
    ◦ Planning/Engaging
    ◦ What were the goals of your seed and full co‐production projects?
    ◦ How were consumers involved in writing the grant application(s)?
    ◦ What factors influenced your engagement methods and recruitment strategy?
    ◦ Executing/Reflecting and Evaluating
    ◦ What went well or not so well?
    ◦ What adaptations were made during implementation?
    ◦ How effective was the co‐production network in achieving project goals?
    ◦ Sustainability/Translation
    ◦ Have the WHRTN grants contributed to further outputs (e.g., manuscripts, resources, presentations, or new funding)?
    ◦ How likely are you to recommend this grant programme?
    ◦ What were the main benefits and areas for improvement?

## Appendix 2 Supporting participant quotations demonstrating consistency between data and findings.

**Table:**

OUTER SETTING	INNOVATION/INTERVENTION CHARACTERISTICS	PROCESS/IMPLEMENTATION	INNER SETTING	CHARACTERISTICS OF INDIVIDUALS
**External pressures** ‘It is still humans trying to gain, you know, have their research territory and that's understandable because it's such a ferociously competitive area, research. You know, the research dollars are so hard to get’ (Academic lead)	**Relative advantage** ‘Allowing that flexibility to enable the natural kind of evolution that that group takes in their thinking and coming together is important.’ (Academic lead #1).	**Assessing need** ‘So I took a different approach and said, well, I'm just going to use things like Google Analytics to see what people want to know about and what they don't know about. s and context’ (Academic lead #5)	**Culture and Connections** ‘There's always been conversation and communication and connection. So everyone has chance to provide feedback’ (consumer #4).‘There's no levels of tokenism, it's all very authentic and that's what shines through’ (Consumer #5)‘If you go in as a researcher or any sort of clinician, you know, you have to leave all your baggage and all your degrees and everything behind you, and you have to go in with the understanding that people are equal’ (Academic lead #2).	**Leadership** ‘We as a consumer, I think that those insights are very important because we are connected with what is happening on the ground’ (Consumer lead #3). ‘Over time, we've refined leadership to the point where now it's very much equal. And I feel that over time it will become more the community‐centred project than the researchers’. (academic lead #1)
**Local attitudes** ‘This is pretty new because otherwise research from my side has been very colonial…basically mainstream English population white. It is not co‐produced like WHRTN… I think this was the first time ever that something like this was introduced’ (Consumer Lead #4)	**Design** ‘I think that there being funding and resources allocated to start the co‐production process from the grant writing stage was a really, I would say, quite revolutionary concept’ (Consumer #8).	**Planning** ‘I guess you feel a little bit in over your head. And like I said, identifying the roles and responsibilities of everyone has not been — it's still not quite clear for some of the academics’ (Academic lead).‘I think it is really important to have roles and expectations clear. And reminder of, you know, what are we actually trying to do here so that I don't try and take everybody off track because of what I've failed to remember’ (Consumer #6).	**Access to Knowledge** ‘It's really important having a very experienced (consumer), she understands the research process. She brings that consumer perspective, but she's able to communicate that with us very clearly’(Academic lead #6)	**Tension for change** ‘I'd say is that it's a radical shift in power around who is making decisions and how those decisions are being made. And I think that can be really challenging for academics.’ (Academic lead# 3) ‘So it's a bit of a tricky balance in terms of that, making sure that I'm not making decisions for everybody but making sure that we can actually move with the project’ (Academic lead #7).
		**Teaming** Y'ou know, it really needs to be that team‐based, collegial, you know, everyone has an equal voice type approach’ (academic lead)	**Infrastructure and resources** ‘So, we've had to put in a lot of sort of in‐kind support to make the project work. There's been a lot of enthusiasm and support to do, but it's because of the goodwill and support of the people involved that have made that happen’ (Academic lead #1).	
		**Adapting** ‘I guess that's how it's been quite organic…(women) like they've sort of chosen how much they want to be involved and their role you, attend(ing) meetings and they provide feedback’ (Consumer #7)		
